# Analysis of endophytic microbiome dataset from roots of black pepper (*Piper nigrum* L.) cultivated in the Central Highlands region, Vietnam using 16S rRNA gene metagenomic next-generation sequencing

**DOI:** 10.1016/j.dib.2022.108108

**Published:** 2022-03-28

**Authors:** Dinh Minh Tran, Thi Huyen Nguyen, To Uyen Huynh, Tu Oanh Do, Quang-Vinh Nguyen, Anh Dzung Nguyen

**Affiliations:** Institute of Biotechnology and Environment, Tay Nguyen University, Buon Ma Thuot, Dak Lak 630000, Vietnam

**Keywords:** Endophytic microbiome, Black pepper root, 16S rRNA gene metagenomic next-generation sequencing, The Central Highlands region

## Abstract

Vietnam is the most prominent black pepper producer and exporter in the world. In 2020, the cultivated area of black pepper in Vietnam was 132.000 hectares and its production was 270.000 tons, in which the Central Highlands region took about 70% of both the cultivated area and production [1]. Hence, this region is thought to be the capital of black pepper cultivation and production in Vietnam. Numerous researches have investigated biodiversity and collected various beneficial endophytic bacteria from this plant in this region; however, traditional methods only were used to isolate such bacteria [2], [3], [4], [5]. Therefore, these studies have a limitation to providing insight into the profiles of the endophytic microorganism dataset in the black pepper plant. Most recently, our work based on the 16S rRNA gene amplicon sequencing revealed an insight into profiles of microbial diversity and its functionality from the sample collected from a forest in this region; however, that work was just focused on soil microbiome dataset from the dry deciduous dipterocarp forest in Yok Don national park [6]. To our knowledge, a dataset of endophytic microbiome of black pepper plant cultivated in the Central Highlands remains unclear.

This report presents a dataset of the endophytic microbiome from a representative sample combined from five different root samples of black pepper (Vinh Linh local variety) cultivated in the Central Highlands of Vietnam using 16S rRNA gene metagenomic next-generation sequencing. The dataset in this work can provide information on the endophytic microbial diversity and its functionality. It can also be useful for developing cultivation techniques by applying endophytic microbial genetic resources for sustainable black pepper production in the Central Highlands, Vietnam, towards the nutrient need in different stages of development and growth.

## Specifications Table

**Table:** 

Subject	Microbiology: Microbiome
Specific subject area	Metagenomics
Type of data	Figures, Tables, and Fastq files
How the data were acquired	Illumina MiSeq platform
Data format	Raw and Analyzed
Description of data collection	Five root samples of the Vinh Linh local variety of the black pepper (*Piper nigrum* L.) plant were collected from five gardens (a 5-year-old black pepper field) in the Central Highlands and then combined into one representative sample. Total DNA was extracted from the sample, and the 16S rRNA gene metagenomic sequencing was performed using the Illumina MiSeq platform
Data source location	Commune/District/Province: Ea Tieu/Cu Kuin, Dak Lak Region: The Central Highlands Country: Vietnam Latitude and longitude coordinates for collected samples: 12°35′12.18′′N,108°04′53.26′′E
Data accessibility	Data are available at the NCBI with Bioproject PRJNA796696 (https://www.ncbi.nlm.nih.gov/Traces/study/?acc=PRJNA796696)

## Value of the Data

• The data provides information on the endophytic microbiome from the root of black pepper (Vinh Linh local variety) cultivated in the Central Highlands, Vietnam.
• The data could be useful for the comparative analysis of the endophytic and rhizosphere microbiome profiles of Vinh Linh local variety black pepper cultivated in the Central Highlands, Vietnam.
• The data could be useful for the comparative analysis of the endophytic microbiome profiles from the root of Vinh Linh local variety black pepper cultivated in the Central Highlands with those of other regions in Vietnam.
• The data could be valuable for developing cultivation techniques by applying endophytic microbial genetic resources for sustainable Vinh Linh local variety black pepper production in the Central Highlands, Vietnam, towards the nutrient need in different stages of development and growth.
• The data could be useful for subsequent studies on the conservation of endophytic microbial genetic resources from Vinh Linh local variety black pepper in the Central Highlands, Vietnam.

## 1 Data Description

**Fig. 1:**
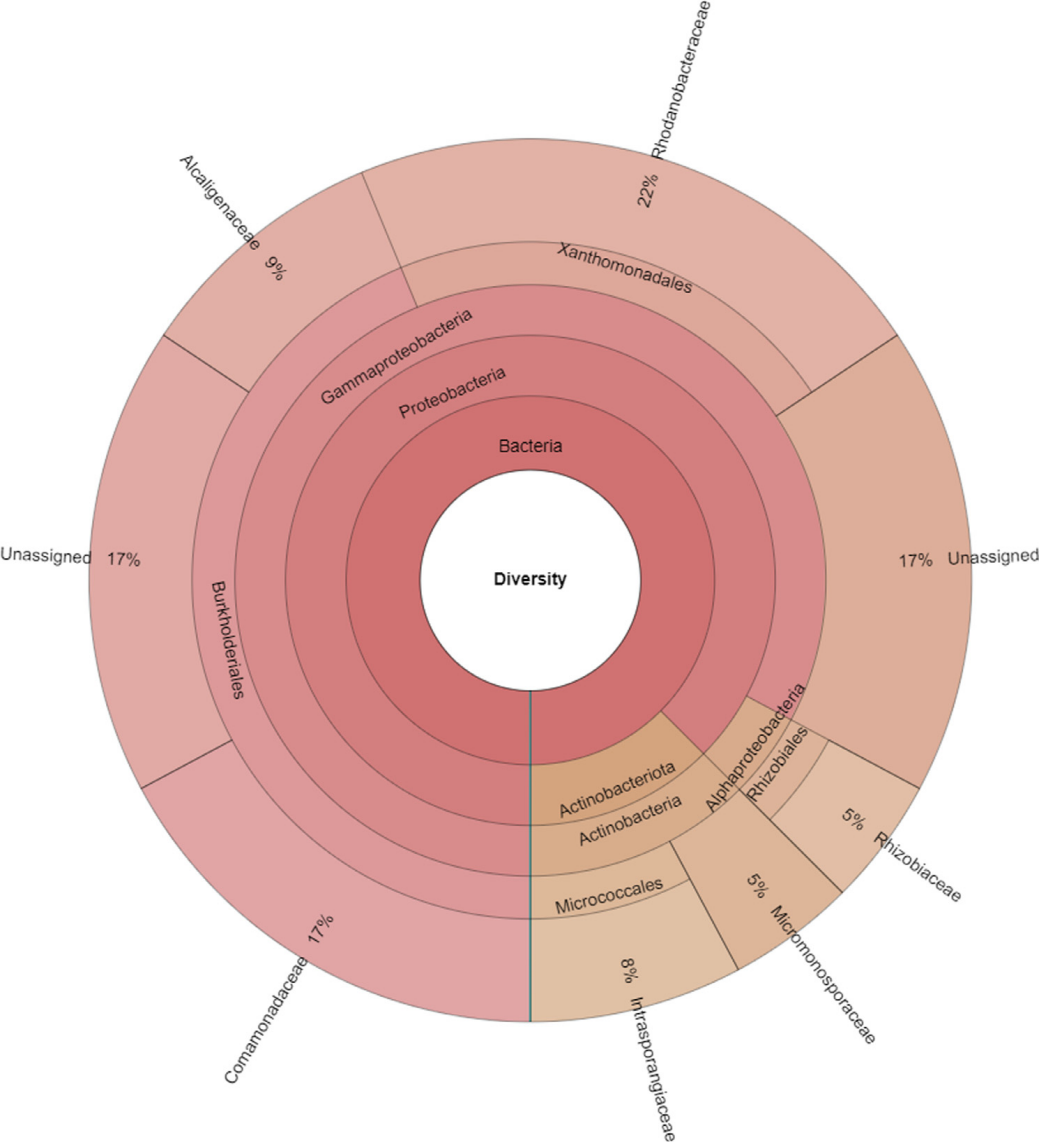
Taxonomic profile of microbiome in the black pepper root sample collected from the Central Highlands region, Vietnam.

The dataset reports the taxonomic and functional profiles of the endophytic microbiome from roots of black pepper cultivated Dak Lak province in the Central Highlands, Vietnam. The result showed that a total of 189,883 reads were classified out of 190,058 analyzed reads (Table 1). As shown in Fig. 1, two phyla, Proteobacteria (87.5%) and Actinobacteriota (12.5%) were identified from the root sample. Three classes were determined; among them, Gammaproteobacteria was 82.8%, Actinobacteria was 12.5%, and Alphaproteobacteria was 4.7%. Among the identified orders, Burkholderiales (43.8%) was found to be the most dominant, followed by Xanthomonadales (21.9%), Micrococcales (7.8%), Micromonosporales (4.7%), and Rhizobiales (4.7%). Moreover, six families (Rhodanobacteraceae, Comamonadaceae, Alcaligenaceae, Rhizobiaceae, Micromonosporaceae, and Intrasporangiaceae) and eight genera (Actinoplanes, Allorhizobium, Neorhizobium, Pararhizobium, Rhizobium, Castellaniella, Dyella, and Rhodanobacter) were identified from the sample.

**Table 1:** Summary statics table.

Reads	Count
Total analyzed reads	190,058
Classified reads	189,883
Unclassified reads	175

**Fig. 2:**
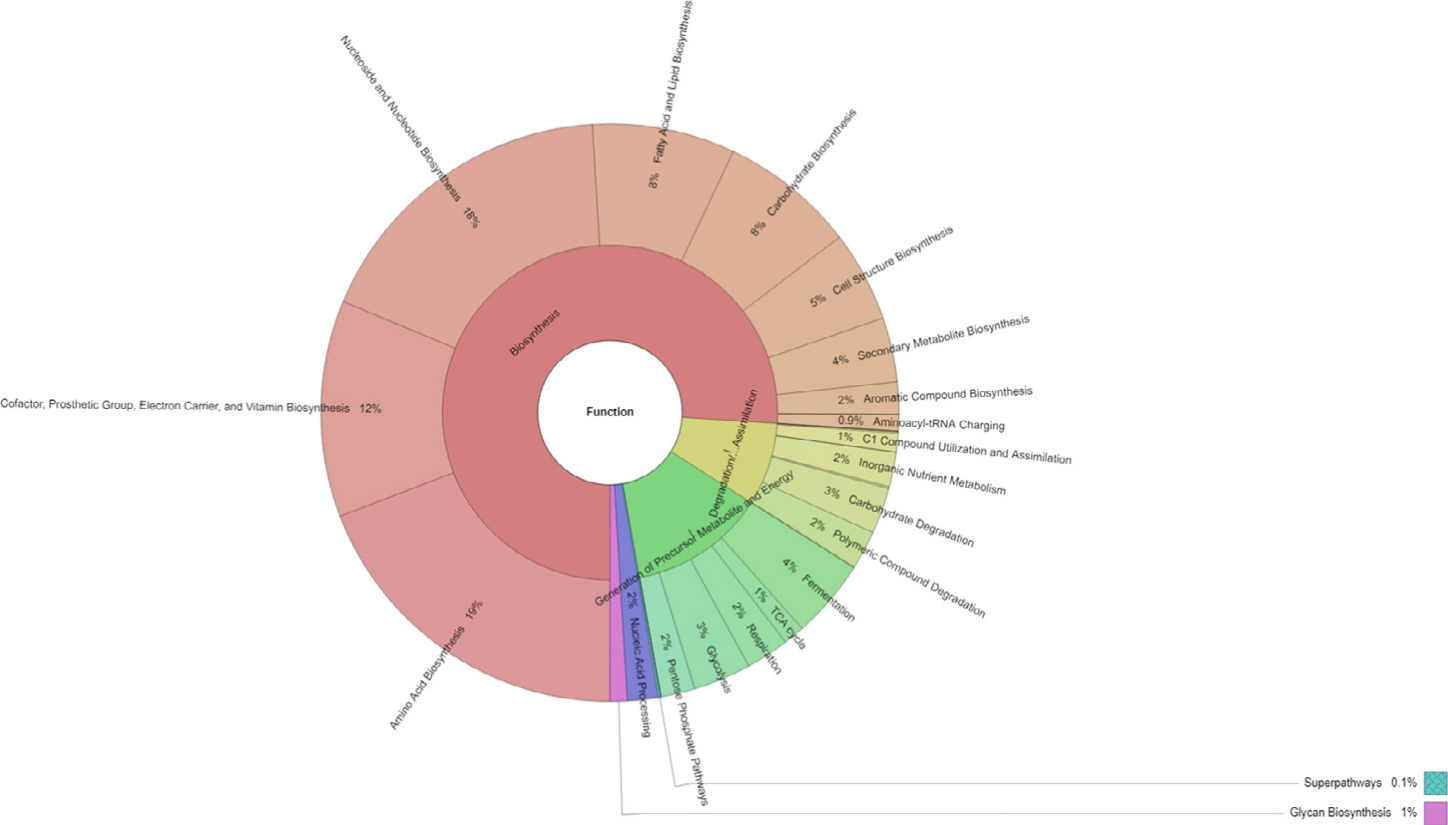
Functional profile of microbiome in the black pepper root sample collected from the Central Highlands region, Vietnam.

As shown in Fig. 2, the main metagenomic function of the microbiome from the black pepper root was biosynthesis (76.03%), followed by the generation of precursor metabolite and energy (13.16%), and degradation/utilization/assimilation (7.99%). Among functions involved in the biosynthesis; amino acid biosynthesis (19.17%) was the most abundant, followed by nucleoside and nucleotide biosynthesis (17.74%); cofactor, prosthetic group, electron carrier, and vitamin biosynthesis (12.11%); fatty acid and lipid biosynthesis (7.99%); carbohydrate biosynthesis (7.59%); cell structure biosynthesis (5.08%); secondary metabolite biosynthesis (3.6%); and nucleic acid processing (1.73%).

## 2 Experimental Design, Materials and Methods

### 2.1 Sample collection

Five root samples (approximately 150 g each) of the black pepper (*Piper nigrum* L.), the Vinh Linh local variety, were collected from five gardens (a 5-year-old black pepper field) in Cu Kuin District, Dak Lak Province, Vietnam, on 30 October 2021, and then combined into one representative sample. The sample was kept in an ice box (4 °C) and transported to the laboratory within one h after sampling. The sample was stored in a low-temperature freezer (−80 °C) until analyzed.

### 2.2 DNA extraction and the 16S rRNA gene amplicon sequencing

DNA extraction and the 16S rRNA gene amplicon sequencing were performed as described previously [6], except for 0.25 g of the sample was used instead of 0.3 g. Briefly, root samples were frozen in liquid nitrogen and total DNA was then extracted from 0.25 g of the sample using the DNeasy PowerSoil kit (Qiagen, Germany). The 16S rRNA gene (regions V1–V9) was then amplified.and according to the supplier's instructions, libraries of the 16S rRNA gene amplicons were prepared using the Swift amplicon 16S plus ITS (internal transcribed spacer) panel kit (Swift Biosciences, USA). Finally, the Illumina MiSeq platform (2 × 150-bp paired ends) was used to perform the 16S rRNA gene amplicon sequencing from the library.

### 2.3 Taxonomic and functional analyses

Taxonomic and functional profiles of microbes identified from the root sample were analyzed as Tran et al. [6] described previously.

## Ethics Statements

None

## CRediT authorship contribution statement

**Dinh Minh Tran:** Conceptualization, Methodology, Investigation, Formal analysis, Software, Data curation, Validation, Visualization, Writing – original draft, Writing – review & editing. **Thi Huyen Nguyen:** Investigation, Formal analysis. **To Uyen Huynh:** Investigation, Formal analysis. **Tu Oanh Do:** Investigation, Formal analysis. **Quang-Vinh Nguyen:** Investigation, Formal analysis. **Anh Dzung Nguyen:** Writing – original draft.

## Declaration of Competing Interest

The authors declare that they have no known competing financial interests or personal relationships that could have appeared to influence the work reported in this paper.

## Data Availability

Black pepper microbiome dataset (Original data).
